# Enhancing Medical Interview Skills Through AI-Simulated Patient Interactions: Nonrandomized Controlled Trial

**DOI:** 10.2196/58753

**Published:** 2024-09-23

**Authors:** Akira Yamamoto, Masahide Koda, Hiroko Ogawa, Tomoko Miyoshi, Yoshinobu Maeda, Fumio Otsuka, Hideo Ino

**Affiliations:** 1 Department of Hematology and Oncology, Okayama University Hospital, Okayama, Japan Okayama Japan; 2 Co-learning Community Healthcare Re-innovation Office, Graduate School of Medicine, Dentistry and Pharmaceutical Sciences, Okayama University, Okayama, Japan Okayama Japan; 3 Department of Primary Care and Medical Education, Dentistry and Pharmaceutical Sciences, Okayama University Graduate School of Medicine, Okayama, Japan Okayama Japan; 4 Department of General Medicine, Okayama University Graduate School of Medicine, Dentistry and Pharmaceutical Sciences, Okayama, Japan Okayama Japan; 5 Center for Education in Medicine and Health Sciences, Okayama University Graduate School of Medicine, Dentistry and Pharmaceutical Sciences, Okayama, Japan Okayama Japan

**Keywords:** medical interview, generative pretrained transformer, large language model, simulation-based learning, OSCE, artificial intelligence, medical education, simulated patients, nonrandomized controlled trial

## Abstract

**Background:**

Medical interviewing is a critical skill in clinical practice, yet opportunities for practical training are limited in Japanese medical schools, necessitating urgent measures. Given advancements in artificial intelligence (AI) technology, its application in the medical field is expanding. However, reports on its application in medical interviews in medical education are scarce.

**Objective:**

This study aimed to investigate whether medical students’ interview skills could be improved by engaging with AI-simulated patients using large language models, including the provision of feedback.

**Methods:**

This nonrandomized controlled trial was conducted with fourth-year medical students in Japan. A simulation program using large language models was provided to 35 students in the intervention group in 2023, while 110 students from 2022 who did not participate in the intervention were selected as the control group. The primary outcome was the score on the Pre-Clinical Clerkship Objective Structured Clinical Examination (pre-CC OSCE), a national standardized clinical skills examination, in medical interviewing. Secondary outcomes included surveys such as the Simulation-Based Training Quality Assurance Tool (SBT-QA10), administered at the start and end of the study.

**Results:**

The AI intervention group showed significantly higher scores on medical interviews than the control group (AI group vs control group: mean 28.1, SD 1.6 vs 27.1, SD 2.2; *P*=.01). There was a trend of inverse correlation between the SBT-QA10 and pre-CC OSCE scores (regression coefficient –2.0 to –2.1). No significant safety concerns were observed.

**Conclusions:**

Education through medical interviews using AI-simulated patients has demonstrated safety and a certain level of educational effectiveness. However, at present, the educational effects of this platform on nonverbal communication skills are limited, suggesting that it should be used as a supplementary tool to traditional simulation education.

## Introduction

Medical interviews play a crucial role not only in the diagnostic process with patients but also in building trust and rapport [1]. Medical interviewing skills are necessary in medical practice and are categorized in the Japanese Model Core Curriculum for Medical Education under the categories of “Comprehensive Patient and Community Perspective” and “Clinical Competencies for Patient Care” [2]. In Japan, the Pre-Clinical Clerkship Objective Structured Clinical Examination (pre-CC OSCE), provided by the Public Interest Incorporated Association, Common Achievement Tests Organization, assesses fourth-year medical students for their competence and aptitude to participate in clinical clerkships [3]. This examination evaluates basic clinical skills, including medical interviewing. It is a nationwide standardized test with very limited flexibility in terms of feedback and the examination itself. Upon passing, medical students are expected to acquire the skills to conduct medical interviews through proper communication and gather necessary information before graduation through participatory clinical clerkships. The standard practice method involves learning medical interviewing in lectures, followed by practice sessions under the supervision of instructors and simulated patients [4].

However, opportunities for Japanese medical students to practice medical interviewing within the medical education curriculum are limited [5]. Japanese medical education has evolved by following the German model since the mid-19th century and the American model since the mid-20th century. As a unique development in Japan, standard curricula and nationwide common exams, including the pre-CC OSCE, have been introduced in medical schools across Japan, aiming to standardize medical education over the past quarter century. However, this has also restricted the autonomy of each university. The learning methods remain predominantly lecture-based and more flexible. In contrast, clinical-based learning methods such as problem-based learning and team-based learning have not yet been widely adopted in Western countries. Even after clinical clerkships, there are many restrictions on medical practice involving patients. This can be attributed to the fact that mandatory clinical training after graduation was implemented much later in Japan than in Western countries, and the integration between undergraduate medical education and postgraduate education is still underdeveloped. Furthermore, simulation education is effective across many fields for learners, not just medical interviewing, but the opportunities to use such education are limited in terms of both location and time [6]. Additionally, from educators’ perspective, introducing medical interview education through simulation faces numerous barriers, including a lack of tutors, staff, simulated patients (including mannequins), and budget constraints [7].

Since the release of ChatGPT by OpenAI in the fall of 2022 [8], generative artificial intelligence (AI) technologies such as large language models (LLMs) have undergone rapid evolution and have been applied across various fields. In the medical domain, their integration is being considered in both clinical and research contexts [9]. One study demonstrated that LLMs can accurately answer questions of the United States Medical Licensing Examination (USMLE), demonstrating their use in medical education and assessment [10]. The COVID-19 pandemic accelerated the digital transformation from traditional bedside teaching to simulation education, including research into remote education models using chatbots [11,12]. However, research integrating LLMs into simulation education remains in its developmental phase [13].

In the field of medical interviewing, a survey of 3018 medical students revealed mixed feelings regarding the integration of LLMs. While some expressed concerns that it might deteriorate the patient-physician relationship, others were hopeful about the potential of AI technology in education, recognizing its dual value [14]. LLMs, which are distinct from previous deep learning–based algorithms, can predict the likelihood of a sequence of words based on the context of the preceding words. Natural and meaningful language sequences can be generated by learning from sufficient textual data. This capability led us to consider their application in practicing medical interviews.

In response to new advances in AI technology and the ongoing digital transformation and to alleviate the lack of educational resources for medical interview training, our team designed a simulation program to improve students’ medical interview skills. This program uses GPT-4 Turbo to fulfill 2 roles: simulated patients and instructors providing feedback. To assess the educational impact of AI-assisted medical interview training on novice learners, specifically fourth-year medical students, we compared the scores from the clinical skills examination, pre-CC OSCE, between the control group, which practiced medical interviews only through traditional methods under the supervision of simulated patients and instructors, and the AI group, which received additional training through AI-simulated patient interviews. Since the medical students were preclinical clerkships, it was not possible to directly measure clinical competence. However, the pre-CC OSCE has shown a significant correlation with performance during clinical clerkships in Japanese medical student cohorts [15]. Notably, the scores of medical interviews have been identified as crucial predictors of performance during clinical clerkships. Therefore, in this study, the analysis was conducted using the scores from medical interviews.

## Methods

### Ethical Considerations

This educational research was approved by the institutional review board of Okayama University (2312-006). In this study, all data were anonymized and deidentified to ensure the privacy and confidentiality of the participants. No personally identifiable information was retained, and appropriate measures were taken to safeguard the participants’ information. Furthermore, no compensation was provided to the participants for their involvement in the research.

### Recruitment

As of November 2023, 35 fourth-year medical students at Okayama University, a national university in Japan, who consented to participate and had completed medical interview practices at least once using our developed AI-simulated patient were designated as the intervention group (AI group, n=35). Fourth-year medical students from Okayama University as of November 2022 who had only a traditional educational program and did not participate in the intervention were selected as the control group (control group, n=110). The practice period was set to 1 month, and the students were provided with an educational environment that allowed them to practice at any time using their laptops or smartphones. After this 1-month training period, the students underwent the pre-CC OSCE, which served as the primary evaluation metric.

### Educational Platforms

The responses of the AI-simulated patients were powered by GPT-4 Turbo, released in November 2023. We integrated it with the service “miibo” (miibo Corporation) through an application programming interface, which allows conversations with specified generative AI in a chat format. In this service, learners cannot see the prompts but can interact with fixed texts, such as case selections and questionnaires that do not involve AI, and choose from options and branch scenarios. While miibo is accessible via a web browser, it was also linked with LINE (LINE Corporation), which is widely used among students in Japan, for enhanced usability and to allow them to practice medical interviews via LINE as well. Learners could conduct interviews in chat format on either platform.

The GPT prompts were primarily composed of 3 elements: basic structure, case information, and feedback. The basic structure designated GPT-4 to act as the simulated patient and the learner as the physician practicing medical interviewing, with the emotional parameters fluctuating in response to the physician’s statements. All outputs were in Japanese. The emotional parameters were set from 1 to 10 for 8 emotions—joy, sadness, anticipation, surprise, fear, disgust, trust, and anger—based on Ekman et al’s [16] theory and Plutchik’s [17] work. Initially, we loaded the case information into ChatGPT-4, ran a common prompt 3 times to estimate the initial emotional parameters, and set the average values. Case information included basic patient details, such as name, age, date of birth, and sex, along with relevant medical history. We prepared cases based on 8 primary symptoms, namely chest pain, abdominal pain, cough, heartburn, fatigue, fever, dizziness, and shortness of breath, which were developed and revised by multiple specialists. The feedback prompt was designed to provide feedback on general communication skills, elicitation of medically important information, and changes in patient emotions based on the conversation logs after the start of the medical interview. An example of a GPT prompt set on miibo is shown in Multimedia Appendix 1.

**Figure 1:**
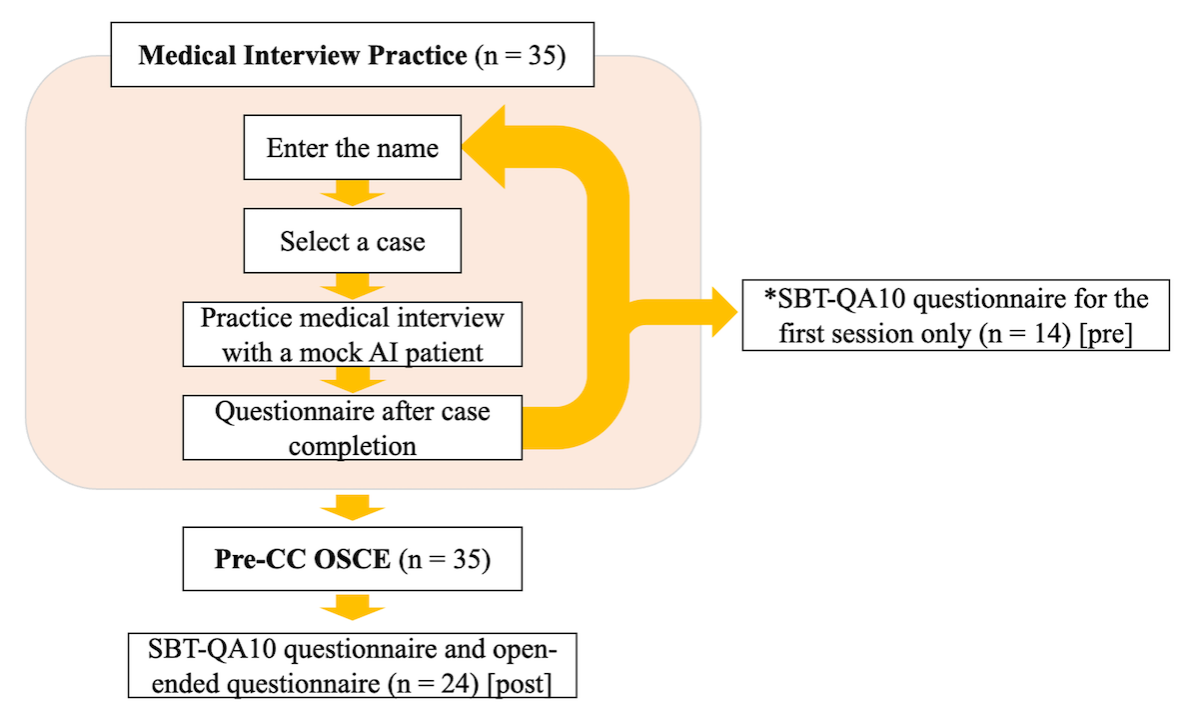
Research overview diagram. Pre-CC OSCE: Pre-Clinical Clerkship Objective Structured Clinical Examination.

Consenting students could access the miibo platform page or a dedicated LINE account via a specified URL, where they could enter their name and select a case. After case selection, they were presented with a scenario starting with the patient entering the consultation room and initiating a greeting, marking the beginning of the medical interview. The conversations were primarily text-based, although voice input was also possible. After completing the medical interview practice, the session could be ended by clicking a button on the screen labeled “End medical interview” or by declaring it, followed by a transition to feedback within the miibo scenario. After the feedback, the conversation log was deleted, and the session proceeded to a questionnaire. After completing the questionnaire, participants were redirected to the case selection section, allowing them to repeat the practice of medical interviews as many times as they desired (Figure 1).

### Questionnaire

After completing the case, the questionnaire asked participants to rate the difficulty of the case on a 5-point scale and assess the realism of the AI-simulated patient, the sense of presence (interaction through emotions with the AI-simulated patient), and their levels of tension and anxiety on a 10-point scale. Participants were also asked to provide open-ended feedback on what they found good and bad about the experience. After completing the first practice session, students were asked to complete a questionnaire based on the Simulation-Based Training Quality Assurance Tool (SBT-QA10, prequestionnaire) [18] to evaluate the quality of the simulation training program. The SBT-QA10, a conventional evaluation tool for simulation training, was not used directly in this study but was partially modified to meet our specific needs. The item “I felt part of the team” was revised to reflect the sense of inclusion within a medical team comprising faculty members.

Additionally, while the medical interview practice solely involved conversations with AI, without direct visibility of faculty, all interaction logs were meticulously reviewed, and responses to questions were managed by faculty. Therefore, items related to support and interaction from faculties were retained. This questionnaire was administered again at the end of the study (postquestionnaire) by gathering open-ended feedback on the overall positive and negative experiences throughout the study.

### Statistical Analysis

The primary outcome measure was the scores related to medical interviewing in the pre-CC OSCE. The pre-CC OSCE consisted of 2 evaluation formats: an overall performance evaluation (summary evaluation, scored from 1 to 6 points) and a score assessment based on individual skills according to a checklist (total score evaluation, scored from 1 to 31 points), both of which were targeted for assessment. As secondary outcome measures, we evaluated the SBT-QA10 and postcase practice questionnaires, specifically assessing the difficulty of the case, the realism of the simulated patient, interaction through emotions, and levels of anxiety and tension. The conversation logs from each practice session were also reviewed. At the start of practice, a unique ID was generated for each device and browser. This ID allowed for the accurate tracking of individual activity records when cross-referenced with the participant’s initial name entry.

Statistical analysis was performed using Prism 9 for macOS (Version 9.5.1). The scores from the pre-CC OSCE were treated as interval data. In addition to the open-ended responses, the questionnaire used a Likert scale. The Mann-Whitney *U* test was used to compare 2 unrelated groups. Fisher exact test was applied to compare sex ratios, and Student *t* tests were used to compare backgrounds between groups based on grade point average (GPA; scored from 0.5 to 4.5). Multiple regression analysis was conducted with the pre-CC OSCE scores as the dependent variable and the questionnaire items as independent variables. The interpretation of correlation coefficients in this study follows the guidelines established by Hinkle et al [19]. According to their criteria, the strength of the correlation is categorized as follows: negligible (0.00-0.30), low (0.30-0.50), moderate (0.50-0.70), high (0.70-0.90), and very high (0.90-1.00). Missing values in the questionnaire items were excluded from the analysis. Additionally, only responses from participants who completed both the pre- and post-SBT-QA10 questionnaires were included in the analysis. The study was conducted with a feasible number of cases, and the effect size was evaluated by calculating Cohen *d* effect size using the pre-CC OSCE scores [19,20].

## Results

Finally, the AI group that received LLM-based simulation education consisted of 35 of 87 students who had consented to participate in this study. In contrast, the control group comprised 110 students who had an opportunity to decline participation, but none chose to refuse. The effect size was calculated using the actual sample size and pre-CC OSCE scores, which revealed 0.48.

No significant differences were observed in the AI and control groups in the age, sex, or GPA of medically related subjects (Table 1). Regarding the medical interview practice, Multimedia Appendix 2 shows an abbreviated version of a representative conversation log and AI feedback, translated from Japanese to English.

**Table 1:** Background.

	AI^a^ group	Control group	*P* value
Sex (female:male), n	15:20	34:76	.11^b^
Age (years), median (IQR)	22 (1)	23 (1)	.37^c^
GPA^d^, mean (SD)	2.9 (0.5)	2.7 (0.6)	.10^e^

^a^AI: artificial intelligence.

^b^Fisher exact test was used for the sex ratio.

^c^The Mann-Whitney *U* test was used for the age.

^d^GPA: grade point average.

^e^The Student *t* test was used for the GPA (scored from 0.5 to 4.5) analysis.

**Figure 2:**
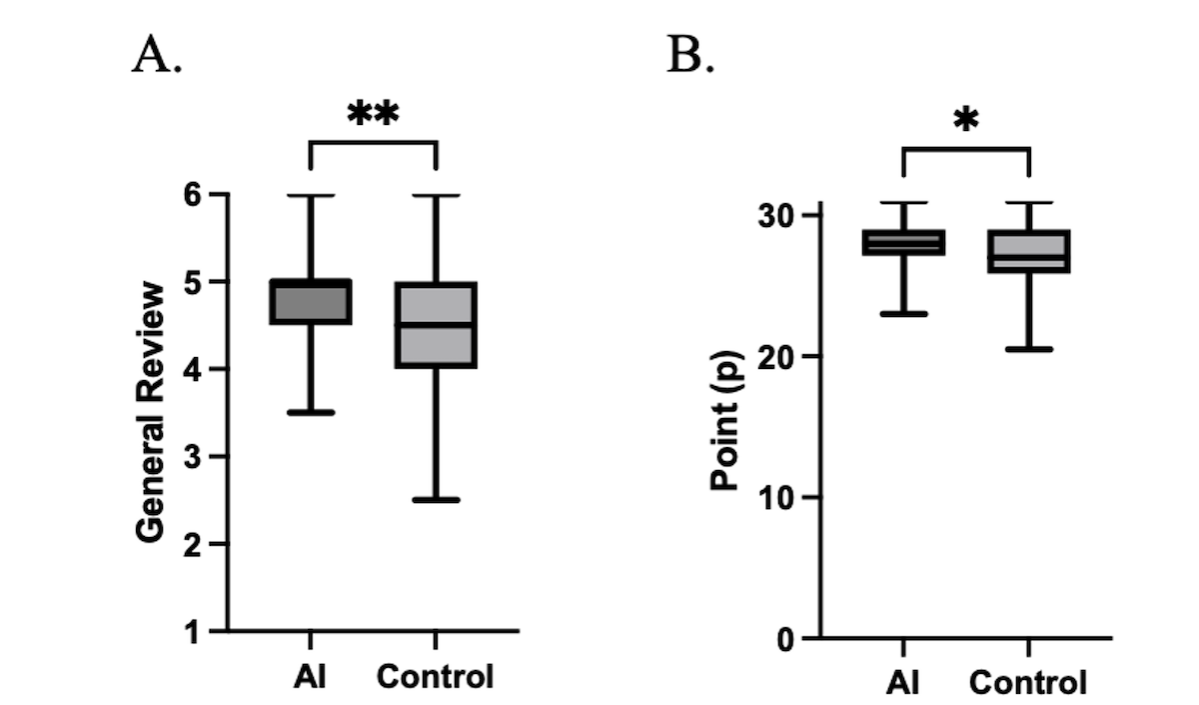
Pre-CC OSCE and LLMs-educational interventions. (A) Summary evaluation (maximum of 6 points, minimum of 1 point on a scale of 1-6). (B) Total score evaluation (maximum 31 points, minimum 0 points graded). Data were analyzed using the Mann-Whitney U test. LLM: large language model; pre-CC OSCE: Pre-Clinical Clerkship Objective Structured Clinical Examination. *P<.05, **P<.001.

Regarding the evaluation of educational effects, when comparing the scores for medical interviews in the pre-CC OSCE, the AI group scored significantly higher than the control group in both summary evaluations (AI vs control: 4.8, SD 0.7 vs 4.5, SD 0.7; 2-tailed; *P*=.007; maximum of 6 points, minimum of 1 point on a scale of 1-6) and total score evaluation (AI vs control: 28.1, SD 1.6 vs 27.1, SD 2.2; 2-tailed; *P*=.01; maximum 31 points, minimum 0 points graded; Figure 2). Additionally, the passing score for the pre-CC OSCE has not been disclosed.

**Figure 3:**
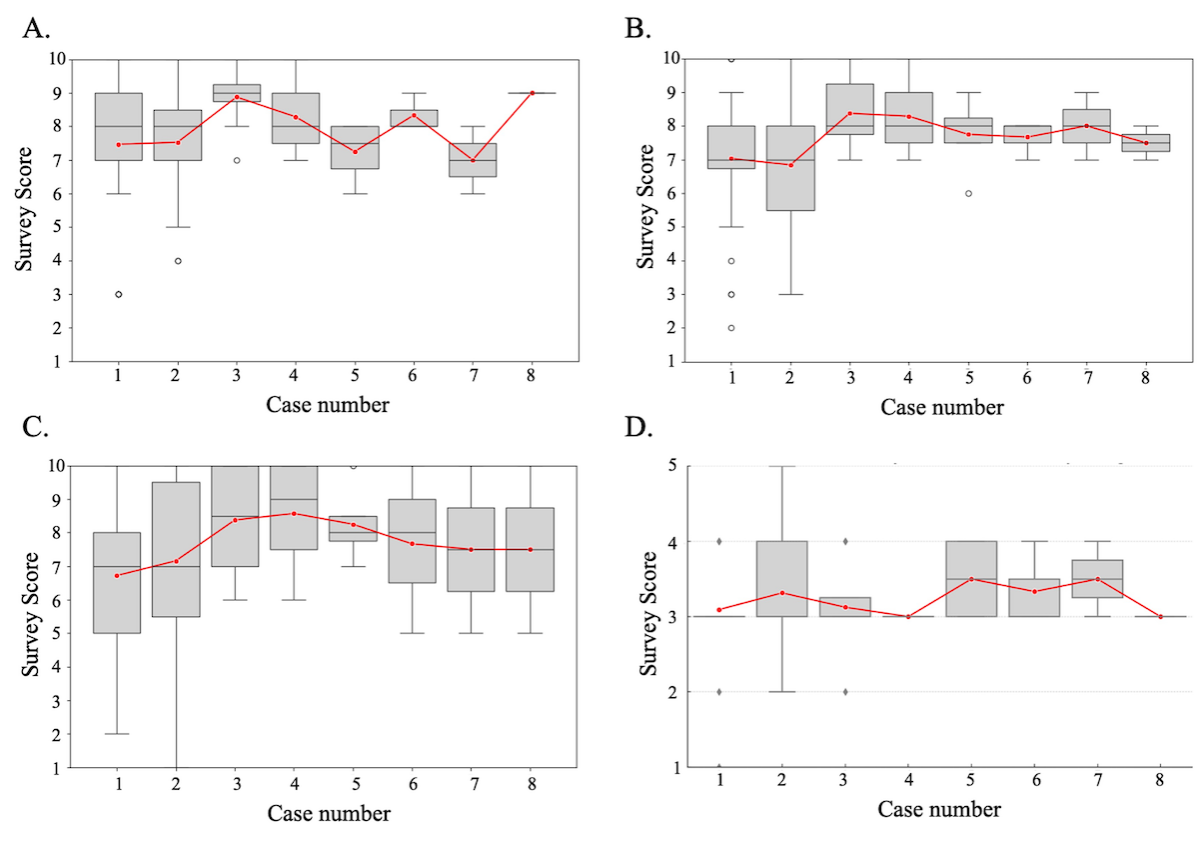
Questionnaire to be taken at the end of each case. (A) Artificial intelligence–simulated patient reproducibility is rated on a scale of 1–10, with 10 indicating “Very High Accuracy” and 1 indicating “no reproduction.” (B) Interaction through emotions is rated on a scale from 1 to 10, where 10 signifies “Very Effective” and 1 signifies “Not Effective at All.” (C) Anxiety and nervousness are rated on a scale from 1 to 10, with 10 indicating “Not Felt at All” and 1 meaning “Felt Very Strongly.” (D) The difficulty of the case is rated on a scale from 1 to 5, where 1 represents “Very Easy” and 5 represents “Very Difficult.”.

The questionnaire results for each case regarding the realism of the AI-simulated patient, interaction through emotions, levels of anxiety and tension, and difficulty of the case are shown in Figure 3. The responses regarding the AI-simulated patients’ reproducibility and interaction through emotions remained stable throughout, with median scores ranging from 7 to 9 for reproducibility and 7 to 8 for emotional interaction. Regarding the levels of anxiety and tension, it was observed that participants experienced them to some degree but without significant stress. Lastly, for the case difficulty, 75%(n=24) of the responses indicated it was “appropriate,” 19%(n=6) found it “difficult,” 3%(n=2) each considered it “easy” and “very easy,” and 0% (n=0) found it “very difficult” in the first instance of the case (n=32). The response “appropriate” was the most common throughout the entire training period, ranging from 50% to 100%.

The scores for the SBT-QA10 in both the pre- and postquestionnaires were relatively high across all items, with the median scores ranging between 4 and 5 (Table 2). No significant changes were observed across all items from pre-questionnaire to postquestionnaire. Additionally, this analysis focused on the group (n=10) that responded to both the pre- and postsurveys. The results from separate analyses for the groups that only responded to the presurvey (n=14) and the postsurvey (n=24) are presented in Multimedia Appendix 3. No significant changes in trends were observed among these groups.

**Table 2:** Evaluation of the simulation program by SBT-QA10^a^.

	SBT-QA10 questionnaire after the first session (pre), median (IQR)	SBT-QA10 questionnaire after pre-CC OSCE^b^ (post), median (IQR)	*P* value (Wilcoxon test)
I felt part of the team (medical stuff team, including faculty)	4.0 (1.3)	4.0 (1.0)	.13
The faculty member(s) interacted well with me	4.5 (1.0)	4.0 (1.0)	.50
Being observed did not intimidate me	4.0 (2.3)	5.0 (2.0)	.50
I felt I was able to act as independently as I wanted to	4.0 (1.0)	4.0 (1.0)	>.99
I felt adequately supported by the faculty member(s)	4.0 (1.0)	4.0 (0.3)	.63
I felt that the scenario was realistic	5.0 (1.0)	4.5 (1.0)	>.99
I understood the purpose of the scenario	4.0 (1.3)	4.5 (1.0)	.38
It did not require a lot of mental effort to play my role in the scenario	4.0 (2.3)	4.0 (2.3)	>.99
I was not distracted by non-relevant objects and events during the scenario	4.0 (1.3)	4.0 (1.5)	>.99
I was focused on being involved in the scenario	4.5 (1.0)	4.5 (1.0)	>.99

^a^SBT-QA10: Simulation-Based Training Quality Assurance Tool. The results of the SBT-QA10 administered after the first session (pre) and pre-CC OSCE (post) for a sample size of 10 are presented for each item. Before-and-after comparisons were analyzed using the Wilcoxon test.

^b^Pre-CC OSCE: Pre-Clinical Clerkship Objective Structured Clinical Examination.

**Figure 4:**
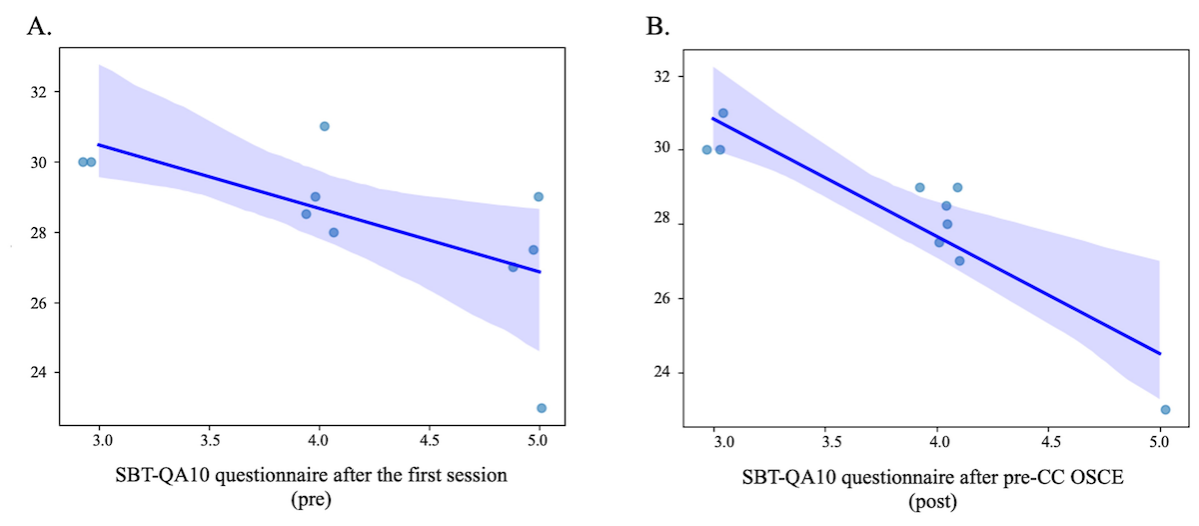
Multiple regression analysis of pre-CC OSCE and SBT-QA10. Multiple regression analysis of the “I felt part of the team (medical stuff team, including faculty)” item in the SBT-QA10 questionnaire. (A) Pre. (B) Post. Pre-CC OSCE: Pre-Clinical Clerkship Objective Structured Clinical Examination; SBT-QA10: Simulation-Based Training Quality Assurance Tool.

Next, we evaluated the group that received AI education to determine which subgroup achieved higher scores on the pre-CC OSCE. Given the high correlation coefficient of 0.75 between the total score evaluation and summary evaluation of the pre-CC OSCE and considering multicollinearity, we focused solely on the total score evaluation for further analysis, incorporating various questionnaire items, GPA and age in a multiple regression analysis. Among these, a consistent trend was observed with the SBT-QA10, where many items showed a negative correlation with the pre-CC OSCE scores. Specifically, the item “I felt part of the team” showed this trend statistically significant in both pre- (coefficients –1.8, SE 0.77; *P*=.047; *R*^2^=0.41) and postevaluations (coefficients –3.2, SE 0.54; *P*<.001; *R*^2^=0.81; Figure 4). When analyzing the total scores of each item in relation to the pre-CC OSCE scores to illustrate the overall trend, a negative correlation was observed; however, none were statistically significant. The analysis results of the combined pre- and post-SBT-QA10 scores are presented in Table 2, including the items of “I felt part of the team” (Table 3). In addition, the results from separate analyses for the groups that only responded to the presurvey (n=14) and the postsurvey (n=24) are presented in Multimedia Appendix 4. The multiple regression analysis revealed consistent negative trends across both the excluded groups and the entire dataset. No significant differences in all the items were observed, including the item ‘I felt part of the team’ in the pre-and postsurveys.

**Table 3:** Multiple regression analysis for analyzing pre-CC OSCE^a^ scores and SBT-QA10^b^.

Item	Const				Independent variable				*R* ^2^		Adjusted *R*^2^
	*β* (SE)	*t* test (*df*)	*P* value	*β* (SE)		*t* test (*df*)	*P* value				
Pre-1: “I felt like part of the team”	35.9 (3.3)	11.0	<.001	–1.8 (0.8)		–2.4	.047	0.41		0.33	
Post-1: “I felt like part of the team”	40.3 (2.1)	19.3	<.001	–3.2 (0.5)		–5.8	<.001	0.81		0.79	
Total scores of pre-SBT-QA10	36.7 (6.9)	5.3	<.001	–2.0 (1.6)		–1.2	.25	0.16		0.05	
Total scores of post-SBT-QA10	37.1 (7.1)	5.3	<.001	–2.1 (1.7)		–1.3	.25	0.16		0.06	

^a^pre-CC OSCE: Pre-Clinical Clerkship Objective Structured Clinical Examination.

^b^SBT-QA10: Simulation-Based Training Quality Assurance Tool. Scores showed an overall inverse correlation with pre-CC OSCE scores, whereas the other items did not show a consistent trend. To illustrate the overall trend, the combined total scores of the SBT-QA10 from both pre and post are presented, including the item of “I felt part of the team” (pre 1 and post 1), in terms of which a significant difference was observed.

The AI group provided detailed feedback on both the advantages and disadvantages of the simulation system, summarized as follows:

- Positive aspects Practicality of training with AI: Participants could practice realistically with an emotional AI, akin to interacting with an actual patient. Convenience and accessibility: Training was available on an easy-to-use platform such as LINE, allowing participants to practice alone without a supervisor and offering flexibility in time and frequency of practice. Increased confidence through practice: Participants gained an understanding of the flow of a medical interview and learned essential questions relevant to clinical settings. Educational value and skill improvement: The training provided practical experience in medical interviewing and valuable feedback that helped improve skills, teaching participants how to inquire in various clinical situations.
- Negative aspects Dialogue and communication with AI: Participants encountered unnatural responses, such as repetitive expressions like “I’m worried” and instability in feedback. Technical and functional aspects: Issues included typing and response time delays, system errors, and operational inconveniences like incorrectly sent messages. Participants suggested that incorporating voice input might improve the experience. Comparison of medical interviews with pre-CC OSCE: Differences were noted in the information provided to simulated patients compared to pre-CC OSCE settings. Some participants appreciated the AI’s superior conversational abilities, whereas others found the AI’s casual speaking manner distracting.

## Discussion

This study is the first to quantitatively verify the effectiveness of entrusting all aspects of medical interview education to AI, from acting as simulated patients to providing feedback as evaluators. It was found that the AI group, for which medical interview practice by LLM-based simulated patients was added to traditional medical interview education when practicing with simulated human patients, scored higher in the pre-CC OSCE medical interviews compared to the control group that only practiced with simulated human patients [4].

As previously reported, medical students do not resist the use of AI in medical education [21], which was evident in this study. The educational style of this study, which allowed students to practice using their smartphones and PCs, enabled them to practice repeatedly at their convenience, as mentioned in the open-ended feedback. This measure not only improved medical interview skills but also reduced anxiety due to a lack of practice and enhanced self-efficacy, suggesting a positive impact on the examination results. Although the 2 groups were from different academic years and might have confounding background factors, basic information such as GPA remained consistent between the groups. This educational method supported by LLMs has the potential to reduce financial and time costs for instructors and simulated patients. This study demonstrates that incorporating this method can effectively supplement the existing shortcomings in medical interview education, thus proving beneficial. However, there are limitations as outlined below. While improvements and applications are anticipated in the future, currently, platforms like this LLM-based medical interview practice should be cautiously used as supplementary tools to traditional simulation education.

### Evaluating Clinical Significance

To verify the statistical significance of this study, the effect size was examined and found to be a moderate effect size [20,22]. Additionally, the minimal clinically significant difference (MCID), an indicator that represents the slightest change of clinical relevance to patients and health care providers, was used to evaluate the meaningfulness of the pre-CC OSCE scores. Unfortunately, there are limited references available that provide specific scores for setting MCID based on pre-CC OSCE scores. We considered the average score minus one standard deviation of the Match group from the study by Horita et al [23] as a reference value for MCID, which was calculated to be 26.9. Initially presented in percentage form in the source study, this value was converted to a point scale to align with the metrics used in our research. In this study, while the Control group’s average score was approximately equal to the MCID, the Intervention group significantly exceeded this benchmark. This suggests that the intervention could have led to clinically meaningful improvements in pre-CC OSCE scores. However, it is important to note that there are various methods for setting an MCID, and given the limited studies, this should be regarded as only one reference point.

### Association Between Pre-CC OSCE Scores and AI Educational Interventions

When exploring which subgroups within the AI group tended to score higher on the pre-CC OSCE, there was an inverse correlation with the SBT-QA10 scores. Educators used the SBT-QA10 to understand the various perceptions experienced by learners during simulation education. High SBT-QA10 scores are generally thought to reflect positive experiences during simulations, leading to subsequent learning. The overall trend of high scores in this study suggests that the training had a positive impact on learners. However, subgroup analysis revealed results that contradict this implication. Unlike traditional simulation education with human-simulated patients, simulations conducted on one’s smartphone or laptop allow for learning in a mentally safe state, potentially resulting in effortless learning within the comfort zone of students, thereby diminishing its effectiveness [24]. Conversely, for students who felt challenged, this may have created a learning zone that enhanced the learning effect.

Additionally, the SBT-QA10 is based on research in Western cultures, and this study, targeting learners in a Japanese cultural context, may require a different interpretation. People from Asian cultures have been reported to be stricter in self-evaluations. This cultural difference may have influenced the results significantly [25,26]. It is, therefore, considered important to adjust the learning environment, such as the difficulty level of cases, while constantly checking feedback from learners and educational outcomes because a good learning environment can vary among learners. However, there is a possibility that some extreme values are influencing the overall trend, as shown in Figure 4. Furthermore, as demonstrated in Multimedia Appendix 4, changing the comparison group eliminates the statistical significance previously observed, although a consistent negative trend is still evident. This suggests that the reliability of the data may be weak. Therefore, the interpretation of this trend should be approached with caution.

### Fabrications by LLMs

Although concerns about fabrication by LLMs have been raised in various contexts [27], their occurrence in this study was limited, and no expressions deviating from the case settings were observed. During the alpha-testing phase with GPT-3.5 Turbo, fabrications were somewhat common, especially in instances where the AI began playing the role of the doctor instead of the simulated patient early in the conversation. Although modifications to the prompts somewhat mitigated this issue with GPT-3.5 Turbo, the change to GPT-4 and GPT-4 Turbo significantly reduced fabrications to a practical level of improvement [28].

The behavioral anomalies of AI in this study can be summarized as follows: The first concern is violations related to public order and morals based on OpenAI’s guidelines. Upon analyzing the conversation logs, it was evident that the students’ inputs did not contain any issues, indicating that the observed discrepancies were due to inaccuracies in the AI output. As the students were preinformed about the possibility of such errors, they could continue with their medical interviews by starting another consultation, preventing it from becoming a significant issue. The second point is related to fabrication in the feedback. For instance, despite confirming the patient’s date of birth and name, there were a few cases in which the feedback suggested that these were not confirmed. This issue was thought to be caused by the prompts treating “confirming the patient’s date of birth and name” as a continuous stream of information, and it was resolved by breaking down the information into separate elements. While prompt adjustments could improve some aspects, the specifications of GPT, which only allow reference to a certain amount of context window and have a limit on the amount of conversation that can be stored, are also considered to be contributing factors [29].

### Safety

No excessive tension or anxiety associated with learning was observed during the simulations. Furthermore, responses from the GPT throughout the study period did not contain any statements that could harm learners’ safety, and no students reported such concerns.

### Limitations

This study was conducted with voluntary participation in educational research without using more desirable intervention methods, such as randomized controlled trials. The emphasis was on equality of educational opportunities, keeping the opportunities of traditional practice with simulated patients. Although consent was obtained from many students, only some of them actually participated in the medical interview practice sessions. This phenomenon can be attributed to unique cultural factors in Japan. Specifically, Japanese medical students often feel a strong inclination to meet others’ expectations when explaining the research, leading them to provide consent [30]. However, this consent might not always reflect their genuine willingness, resulting in a lower actual participation rate. Consequently, the sample size was limited.

In addition, this study employed LLM-simulated patients’ interventions and evaluated their effectiveness through a simulation-based assessment such as the pre-CC OSCE. However, reports suggest that qualitative improvements in simulators do not directly cause clinical skill enhancement, underscoring the importance of conducting clinical skill assessments in real-world settings as much as possible [31]. As this study focused on pre-clinical clerkship medical students, the assessment was limited to an indirect and short-term evaluation of clinical skills using medical interview scores from the pre-CC OSCE [15]. Therefore, we plan to conduct long-term evaluations of this program for clinical clerkship students and early-career physicians in actual clinical settings in future studies. Moreover, since this platform is text-based, its capacity to handle non-verbal communication is restricted. For instance, similar to how GPT-4 can partially recognize visual and voice information, further advancements in LLM technologies that could better recognize and process human emotions and sensory inputs may help overcome this limitation. Currently, LLM-based medical interview simulation training should serve as a supplemental tool to existing medical interview education and is not yet capable of fully replacing traditional methods. Nonverbal communication skills, which are crucial, are still best developed through instructor-led training involving human-simulated patients. This study was conducted as a pilot project for the future application of LLMs in medical interview training.

### Plan

This study suggests the potential for a significant reduction in the workload of instructors and simulated patients in medical interview practice while maintaining educational effects for medical students. Furthermore, the introduction of LLM-simulated patients to clinical skill examinations such as the pre-CC OSCE is conceivable. It holds promise not only for educating young doctors but also for the lifelong education of doctors, including simulations for handling complex cases in clinical settings. However, when introducing LLM simulations into medical education, caution is necessary regarding ethical considerations and accuracy, as previously pointed out. Completely replacing traditional instructor-led training with AI carries risks, and further studies thereon are required [13,21].

Improvements in prompts and the evolution of AI technology suggest that more realistic and accurate simulation education can be expected in the future. The integration of AI into medical education is inevitable; however, it has the potential to disrupt traditional medical education practices. Educators must remain vigilant regarding the potential positive and negative impacts of this integration [32]. Concurrently, it is essential to continue research on AI-mediated medical education to explore its applicability and limitations.

### Conclusions

Education on medical interviewing using LLM-simulated patients demonstrated superior educational effectiveness while maintaining safety. This platform holds promise for multifaceted applications in the field of medical education in the future. It should be noted that this study only assessed short-term impacts and did not directly evaluate clinical skills. Additionally, due to the extremely limited educational effects on nonverbal communication skills, it is currently advisable to use this platform as a supplementary tool in medical interview training. Given the occurrence of fabrications and the opaque nature of LLM technology across various companies, caution and intense monitoring by tutors are essential when incorporating LLM-based educational platforms into medical education.

## Appendix

Multimedia Appendix 1 Prompt for medical interview practice.

Multimedia Appendix 2 Representative communication log and feedback in medical interview practice.

Multimedia Appendix 3 The simulation program was evaluated using the Simulation-Based Training Quality Assurance Tool, which was conducted separately in the groups that responded to the pre-survey (n=14) and the post-survey (n=24).

Multimedia Appendix 4 Multiple regression analysis for analyzing Pre-Clinical Clerkship Objective Structured Clinical Examination scores and Simulation-Based Training Quality Assurance Tool was conducted separately in the groups that responded to the presurvey (n=14) and the postsurvey (n=24).

## Abbreviations

AI: artificial intelligence
GPA: grade point average
LLM: large language model
MCID: minimal clinically important difference
Pre-CC OSCE: Pre-Clinical Clerkship Objective Structured Clinical Examination
SBT-QA10: Simulation-Based Training Quality Assurance Tool
USMLE: United States Medical Licensing Examination
