# Supplementary material for: Enhancing Medical Interview Skills Through AI-Simulated Patient Interactions: Nonrandomized Controlled Trial
Source: JMIR Med Educ. 2024 Sep 23;10:e58753. doi: 10.2196/58753 (PMC11459107; doi:10.2196/58753)
Supplement: Multimedia Appendix 2 [file mededu_v10i1e58753_app2.docx]

**Case Selection**

AI: Please tell me your name.

Student: My name is (name).

AI: Please choose a scenario below: chest pain, abdominal pain, cough, heartburn, fatigue, fever, dizziness, and shortness of breath.

Student: [Cough]

AI: Then, Dr. (name), let's start the role-play from the point where the patient with [cough] enters the consultation room. First, you, as the doctor, should greet them.

**Case Practice**

Student: I will be in charge of the medical interview today, I am (name), a fourth-year medical student.

AI: Doctor, thank you in advance. Recently, I've been having chest pain and coughing. I'm a bit anxious ...

Student: That must be tough for you. What kind of cough do you have?

AI: It's pretty severe, and since yesterday, I've been coughing up brown phlegm. There was even a bit of blood mixed in, which scared me.

Student: Does it affect your daily life?

AI: Yes, it does. The cough is so bad I can hardly sleep at night, and I feel so lethargic that I might have to miss university.

Student: That sounds very difficult. How severe is the cough compared to coughs you've had before?

AI: Seriously, it's tough. This is the first time I've had such a bad cough. Normally, I don't get this bad even when I catch a cold ...

Student: I see. Do you have any pain?

AI: Yes, my chest hurts. It hurts every time I cough, and I want something to be done about it.

Student: Could you point to where it hurts?

AI: Here, around the middle of my chest is especially painful. It throbs every time I cough ...

Student: Understood. So, it's a throbbing pain. Has the intensity of the pain changed since last night?

AI: No, the pain hasn't really changed since yesterday. But, I feel like the cough and phlegm are getting worse.

Student: Is there anything that makes the symptoms better or worse?

AI: Hmm, nothing really makes it better, but when I take a deep breath or lie down, the cough gets stronger and the pain increases.

Student: Do you have any symptoms other than cough, phlegm, and chest pain?

AI: Yes, I also have a slight headache. And I feel extremely lethargic. Seeing the blood in my phlegm really shocked me ...

Student: You feel lethargic. Do you have a fever?

AI: I haven't measured it, but my body feels feverish, so I probably do have a fever.

[...]

Student: Just to confirm, you are a university student, right?

AI: Yes, that's right. I'm studying at university, and I'm a bit worried right now ... What if it's COVID-19?

Student: It's a worrying time indeed. Regarding your diet, do you have any preferences for what you eat?

AI: I generally eat a variety of foods without much bias. But lately, because of the severe cough, I don't have much appetite.

Student: That makes sense. Have you been able to sleep well since the symptoms appeared?

AI: No, the cough is so bad at night that I can't sleep well. And that makes me very tired during the day ...

Student: I understand. Have you noticed any changes in your weight?

AI: I haven't weighed myself, so I can't say for sure, but my appetite has decreased, so I might have lost some weight.

Student: How about your bowel movements?

AI: No problems there. It's as usual.

Student: Thank you. I would like to ask a bit more about your university life. Is it busy?

AI: Yes, it's quite busy. There are many classes, and preparing for reports and tests is hard. But right now, I'm more worried about my health and can't concentrate much.

Student: That's understandable. Do you live alone?

AI: Yes, I live alone. So, even when I'm feeling like this, I have to manage on my own.

Student: Understood. Besides COVID-19, are there any other diseases you are worried about?

AI: Well, because I coughed up blood, I'm worried it might be some lung disease. Also, since my mother had cancer, that's a bit of a concern too.

Student: Understood. May I ask about any tests or treatments you are hoping for?

AI: Yes, I want to find out the cause of my condition and undergo any necessary tests. I want to get treated and return to my normal life as soon as possible.

Student: Understood. Let me summarize our conversation. Please let me know if there's anything I've missed or you'd like to add. You started coughing two days ago, and last night you coughed up blood-mixed phlegm. In addition to the throbbing chest pain, you feel lethargic and likely have a fever. You want to undergo all necessary tests to address the symptoms. Is that correct?

AI: Yes, that's right. Also, I have a slight headache. I hope to find the cause soon and receive appropriate treatment.

Student: My apologies. Thank you. Now, we'll move on to the physical examination, so please wait in the outer waiting room. Thank you for today. Please take care.

AI: Understood. Thank you for the consultation. I appreciate your help.

Student: [Ends the medical interview].

**Feedback**

AI: Thank you for your effort in the medical interview practice. Now, I will provide feedback on the medical interview.

1. Communication

The patient's name and date of birth were not confirmed. However, respectful language was used towards the patient, and the communication was good. It's also important to actively use words that empathize with the patient's anxiety and pain.

2. Medical Interview Items

The confirmation of symptoms such as cough, loss of appetite, sleep quality, and weight change was appropriate. However, the relevance of the patient's busy university life and living alone to their symptoms was not clear, so a more symptom-focused interview is needed. Asking about diseases the patient worries about besides COVID-19 was good, but questions about desired tests and treatments could be more specific.

3. Patient’s Emotions

The empathy and understanding shown towards the patient's anxiety about COVID-19 were good, and the patient seemed to be comfortable discussing their symptoms and concerns. However, a bit more comforting words before telling the patient to wait for the examination could have provided additional reassurance.

Please keep these points in mind and continue practicing. Press the OK button to continue practicing.

**(Back to Case Selection)**

This Supplemental Text 2. shows representative examples of medical interview practices and feedback from the AI-simulated patients. The GPT prompt is documented in Supplemental Text 1. Although these interactions originally occurred in Japanese, they have been translated into English.
