# Supplementary material for: Enhancing Medical Interview Skills Through AI-Simulated Patient Interactions: Nonrandomized Controlled Trial
Source: JMIR Med Educ. 2024 Sep 23;10:e58753. doi: 10.2196/58753 (PMC11459107; doi:10.2196/58753)
Supplement: Multimedia Appendix 3 [file mededu_v10i1e58753_app3.docx]

|  | SBT-QA10 questionnaire  after the first session  (pre) | |  | SBT-QA10 questionnaire after pre-CC OSCE  (post) | | Wilcoxon test |
| --- | --- | --- | --- | --- | --- | --- |
|  | median | IQR |  | median | IQR | *p* value |
| I felt part of the team (medical stuff team including faculty) | 4.0 | 1.5 |  | 4.0 | 1.0 | .19 |
| The faculty member(s) interacted well with me | 5.0 | 1.0 |  | 4.0 | 0.8 | .31 |
| Being observed did not intimidate me | 4.0 | 2.0 |  | 5.0 | 1.8 | .19 |
| I felt I was able to act as independently as I wanted to | 4.0 | 1.0 |  | 4.0 | 1.0 | >.99 |
| I felt adequately supported by the faculty member(s) | 4.0 | 1.0 |  | 4.0 | 1.0 | .38 |
| I felt that the scenario was realistic | 5.0 | 1.0 |  | 4.0 | 1.0 | .38 |
| I understood the purpose of the scenario | 4.0 | 1.0 |  | 4.5 | 1.0 | .34 |
| It did not require a lot of mental effort to play my role in the scenario | 4.0 | 2.5 |  | 4.5 | 2.0 | >.99 |
| I was not distracted by non-relevant objects and events during the scenario | 4.0 | 1.5 |  | 4.0 | 1.8 | >.99 |
| I was focused on being involved in the scenario | 5.0 | 1.0 |  | 4.0 | 1.0 | >.99 |

The results of the SBT-QA10 administered after the first session (pre) and pre-CC OSCE (post) are presented for each item. Before- and after-comparisons were analyzed using the Wilcoxon test.

SBT-QA10: Questionnaire after the first session (*n* = 14), and after pre-CC OSCE (*n* = 24).

SBT-QA10: Simulation-Based Training Quality Assurance Tool.

IQR: Interquartile Range.
