# Supplementary material for: Enhancing Medical Interview Skills Through AI-Simulated Patient Interactions: Nonrandomized Controlled Trial
Source: JMIR Med Educ. 2024 Sep 23;10:e58753. doi: 10.2196/58753 (PMC11459107; doi:10.2196/58753)
Supplement: Multimedia Appendix 4 [file mededu_v10i1e58753_app4.docx]

|  | Const | | | |  | Independent variable | | | |  |  |  |
| --- | --- | --- | --- | --- | --- | --- | --- | --- | --- | --- | --- | --- |
| Item | *β* | *SE* | *t* | *p* |  | *β* | *SE* | *t* | *p* |  | *R^2^* | Adj *R^2^* |
| Pre-1  “I felt the part of the team” | 31.3 | 2.2 | 14.4 | <.001 |  | -0.8 | 0.5 | -1.6 | .14 |  | 0.17 | 0.10 |
| Post-1  “I felt the part of the team” | 30.3 | 1.3 | 22.8 | <.001 |  | -0.6 | 0.4 | -1.7 | .10 |  | 0.12 | 0.08 |
| Total scores of pre-SBT-QA10 | 31.7 | 3.8 | 8.3 | <0.001 |  | -0.9 | 0.9 | -1.0 | .30 |  | 0.07 | -0.004 |
| Total scores of post-SBT-QA10 | 30.9 | 2.3 | 13.2 | <.001 |  | -0.7 | 0.6 | -1.2 | .23 |  | 0.07 | 0.02 |

Multiple regression analysis of SBT-QA10 and pre-CC OSCE scores was conducted without excluding any samples, analyzing both pre- (n=14) and post- (n=24) surveys.

*β*: Coefficients, *SE*: Standard Errors, *t*: *t* value, *p*: *p* value, *R*^2^: R-squared, Adj *R*^2^: Adjusted R-squared.
